# Fetal Distress as a Determinant for Refeeding Syndrome in Preterm Neonates

**DOI:** 10.3390/nu17091417

**Published:** 2025-04-23

**Authors:** Maria Di Chiara, Caterina Spiriti, Flavia Gloria, Gianluigi Laccetta, Lucia Dito, Magda Gharbiya, Giuseppe Rizzo, Gianluca Terrin

**Affiliations:** 1 Department of Maternal and Child Health, Policlinico Umberto I, Sapienza University, 00161 Rome, Italy; spiriti.1844666@studenti.uniroma1.it (C.S.); gloria.1891102@studenti.uniroma1.it (F.G.); gianluigi.laccetta@uniroma1.it (G.L.); lucia.dito@yahoo.it (L.D.); giuseppe.rizzo@uniroma1.it (G.R.); gianluca.terrin@uniroma1.it (G.T.); 2 Department of Sense Organs, Sapienza University of Rome, 155, Viale del Policlinico, 00161 Rome, Italy; magda.gharbiya@uniroma1.it

**Keywords:** parenteral nutrition, preterm neonates, phosphorus, refeeding syndrome, ROP

## Abstract

**Background/Objectives**: Preterm neonates receiving parenteral nutrition (PN) are at risk of developing refeeding syndrome (RS). Risk factors and the related consequences remain largely undefined. In particular, the reason why only some preterm neonates out of a group receiving the same nutritional protocol will develop RS is yet to be fully understood. The aims of this study were to explore the clinical and nutritional factors contributing to RS and to assess the clinical consequences of this condition. **Methods**: A retrospective study was conducted, including all newborns with gestational age ≤ 34 weeks and/or body birth weight ≤ 1500 g who were consecutively admitted to the neonatal intensive care unit (NICU) of “Umberto I” Hospital, Sapienza University of Rome, from 2015 to 2022. The population was divided into two groups comprising newborns who developed RS (cases) and infants who did not develop the condition (controls) up to the first 2 weeks of life. The enrolled newborns were compared for clinical and nutritional factors and main morbidities. **Results**: A total of 412 neonates were enrolled, consisting of 53 cases and 359 controls. The main prenatal risk factor for RS was found to be fetal distress (*p* = 0.028). The occurrence of RS was identified as statistically significantly associated (*p* = 0.010; *p* = 0.007) with the development of extrauterine growth restriction (EUGR) and retinopathy of prematurity (ROP). **Conclusions**: Fetal distress is the predominant perinatal risk factor associated with the development of RS in preterm neonates managed with early currently recommended PN. These findings suggest an increased risk of ROP and EUGR in preterm neonates with RS.

## 1. Introduction

Preterm neonates frequently fail to achieve expected growth rates when compared to those observed in utero at the same gestational age (GA) and are at high risk of developing increased morbidity compared to neonates at term [1,2].

The optimization of early protein and energy intakes through parenteral nutrition (PN) administration has been thought to be a worthwhile tool to reduce extrauterine growth restriction (EUGR), especially in very-low-birth-weight (VLBW) newborns, to improve clinical outcomes. The actual composition of PN is related to short- and long-term outcomes, such as metabolic complications, that are still under study [3,4].

Over the last years, the composition of PN has led to the adoption of “aggressive” PN, with high concentrations of amino acids, carbohydrates, and lipids. The aim of “aggressive” PN is to ensure adequate extrauterine growth, counteract cellular catabolism, and prevent early nutritional deficits [5]. However, evidence has shown that enhanced early PN, according to the current recommendations, may be associated with potentially harmful effects in preterm newborns [6,7]. The metabolism of phosphorus and calcium, along with their plasma concentrations, may also be influenced by an aggressive nutritional approach. The high and early intake of PN contributes to the shifting of cellular metabolism into an anabolic state. This results in the transfer of phosphate from bones to cells and an increased release of calcium [8,9].

This combination of factors can lead to dramatic variations in serum electrolyte concentrations during early aggressive PN, leading to refeeding syndrome (RS) [10,11]. 

Despite there being no universally accepted definition of RS, with the lack of consensus being particularly evident across different age groups, most studies agree that it is characterized by electrolyte imbalances (hypercalcemia and hypophosphatemia) that occur when nutrition is reintroduced after prolonged malnutrition or starvation. Although hypercalcemia is not a widely recognized feature of refeeding syndrome alone, it has been previously documented in neonates, particularly in preterm infants. Its involvement in the pathophysiology of RS in this population has been explored in several studies. Specifically, hypercalcemia arises because calcium is released from bone, along with the phosphate needed to maintain serum phosphate concentrations during the anabolic phase that characterizes refeeding syndrome, as described by Moltu, Ichikawa, Bonsante, and Cormack [8,12,13].

Even though RS has been documented in both the adult and pediatric populations, its description and related harmful consequences in the neonatal population remain largely undefined. In the current literature involving neonates, the condition is often described as the coexistence of two electrolyte imbalances: hypercalcemia and hypophosphatemia. The reason why only some preterm neonates will develop RS out of a group receiving the same nutritional protocol according to current guidelines is yet to be fully understood. The evidence for predicting RS is poor and marked by inconsistent findings. Therefore, the role of potential clinical risks or protective factors leading to RS in preterm neonates who were managed with currently recommended early PN is still undefined. Moreover, the implications for short- and long-term morbidity outcomes associated with RS are not clearly defined.

We hypothesized that certain pre-perinatal factors might influence which preterm neonates, despite receiving routine PN in accordance with current recommendations, are at risk of developing RS.

Starting from these considerations, the aims of this study were (i) to explore clinical and nutritional factors contributing to the development of RS and (ii) to assess the effects of this condition on the occurrence of short- and long-term morbidity.

## 2. Materials and Methods

### 2.1. Study Design and Population

We designed a retrospective study including all newborns with a gestational age (GA) ≤ 34 weeks and/or body birth weight ≤ 1500 g who were consecutively admitted to the neonatal intensive care unit (NICU) of “Umberto I” Hospital, Sapienza University of Rome, from January 2015 to November 2022.

We excluded neonates with major congenital malformations, inborn metabolism errors, congenital or acquired immunodeficiency, congenital infections, incomplete clinical data, death, or transfer to other hospitals before 72 h of life.

The diagnosis of RS was established by the simultaneous occurrence of hypophosphatemia (P < 1.6 mmol/L) and hypercalcemia (ionized Ca > 1.3 mmol/L), referred to as hypophosphatemic hypercalcemia, up to 2 weeks of life [8,9].

Definitive diagnosis was confirmed after an agreement between three researchers (G.T., M.Di.C., and C.S.). Among the eligible subjects, we selected newborns with RS as cases. In addition, newborns without RS were classified as potential controls.

### 2.2. Nutritional Protocol

All neonates enrolled in this study received PN within 24 h upon admission into the NICU, following the placement of either central vascular access (CVO) or a peripheral (PICC) line. PN was administered to maintain adequate fluids, electrolytes, and nutritional intake until the achievement of full enteral feeding (FEF) (120 kcal/kg/day).

Neonatologists in charge prescribed individualized PN daily based on each neonate’s clinical condition, laboratory results, and weight by using custom-built software (https://drive.google.com/file/d/1IcGKfWGPls7wKg8IaOso8oTVMOmmC3wU/view?usp=sharing (accessed on 6 April 2025), version 2015, last update carried out in 2023). The macronutrient and micronutrient content administered through PN was calculated based on the local nutritional protocol. The calcium infusion (calcium gluconate 10%) starts on day 1 of life at 40 mg/kg/day and is gradually increased until reaching 80 mg/kg/day by the eighth day of life. The phosphorus infusion (sodium glycerophosphate) starts on day 1 of life at 25 mg/kg/day and is gradually increased until reaching 75 mg/kg/day by the eighth day of life. The phosphorus/calcium ratio was 1.

Enteral nutrition (EN) was started with minimal enteral feeding (10–20 mL/kg/day divided into four to eight feeds), which commenced as soon as the general clinical condition was stable. Starting from 48 and 96 h, our protocol recommends increasing the feeds of 15–30 mL/kg/day according to birth weight in otherwise stable infants in the absence of feeding intolerances in the previous 24 h.

### 2.3. Data Collection

Neonatal data from NICU admission until discharge, transfer to another hospital, or death were retrieved and stored in a specific database. Prenatal, perinatal, and postnatal data were reviewed and collected. The term fetal distress refers to the coexistence of fetal Doppler abnormalities and acidosis at birth (umbilical cord pH < 7.2).

Daily data on the intake of PN macro- and micronutrients were also collected with a specific chart by physicians who were unaware of this study’s aims.

Auxological parameters (standardized and unstandardized) were prospectively collected and reviewed. Length at birth was measured from the top of the head to the soles of the feet using a neonatal stadiometer. Head circumference at birth was measured using a non-elastic tape measure. Weight, length, and head circumference percentiles and Z-score by gender were calculated using the Italian Neonatal Study Charts (INeS) growth curves. EUGR was defined as a reduction in the weight Z-score of >1.0 from birth to a postmenstrual age of 36 weeks [14].

Data on the length of stay and main morbidities, such as necrotizing enterocolitis (NEC) stage of Bell ≥ 2 [15], intraventricular hemorrhage (IVH) ≥ 2, periventricular leukomalacia (PVL) [16,17], sepsis (confirmed by positive cultures), retinopathy of prematurity (ROP) ≥ 3 [18], and bronchopulmonary dysplasia (BPD) [19], were collected according to standardized criteria and retrieved. A third-party blind observer not directly involved in NICU patient care and not informed of this study’s aims assessed the confirmed diagnoses.

### 2.4. Ethics

This study was conducted in compliance with the World Medical Association’s Declaration of Helsinki for medical research involving human subjects and was approved by the Ethics Committee of Policlinico Umberto I, Sapienza University of Rome. Written informed consent was obtained from all parents before enrollment. Individual-level data cannot be publicly shared due to privacy laws (Italian Law: D.Lgs. n. 196/2003). Data are available from the Maternal and Child Health Department of Policlinico Umberto I, Sapienza University, Rome.

### 2.5. Statistical Analysis

Statistical analysis was performed using the Statistical Package for Social Sciences Software for Microsoft Windows (SPSS Inc., Chicago, IL, USA), version 27.0. We checked for normality using the Shapiro–Wilk test. Continuous variables were compared using the Student’s *t*-test. The mean and standard deviation, or median and interquartile range, summarized continuous variables. We used the chi-squared test for categorical variables and the Mann–Whitney U test for unpaired variables. The level of significance for all statistical tests was 2-sided (*p* < 0.05). After checking for assumptions, binary regression analysis with a stepwise method was used to study the possible influence of confounding variables that were statistically significant in preliminary analyses (GA, BW, male gender, fetal distress, MEF, protein caloric intake, non-protein caloric intake, calcium, and phosphorus) on the occurrence of RS. Individual binary logistic regression analyses were performed to investigate the influence of covariates on the occurrence of neonatal morbidities that were statistically significant in preliminary analyses. A statistician unaware of this study’s aims received the database in which each patient was associated with a code.

## 3. Results

Among a cohort of 425 premature neonates, thirteen were excluded because of incomplete clinical data (five), congenital malformations (one), or transfer to another hospital (seven). We included 412 neonates in the analysis. We observed 53 neonates who developed RS (cases) and 359 neonates who did not develop the condition (controls). The observed incidence rate of RS was 12.8% in the studied population. However, when considering only newborns who received more than 90% of their total caloric intake through parenteral nutrition (32 cases and 112 controls), the observed incidence rate of RS doubled (22.2%).

The main baseline clinical characteristics, categorized into baseline and nutritional factors of participating cases and controls, are summarized in Table 1 and Table 2.

At baseline, the prenatal characteristics of the patients were similar between the two groups (Table 1). We observed that fetal distress was significantly higher in cases than controls (60.0% vs. 40.0%, *p* = 0.002). Table 3 shows the baseline nutritional factors in neonates enrolled in this study (Table 2).

**Figure 1:**
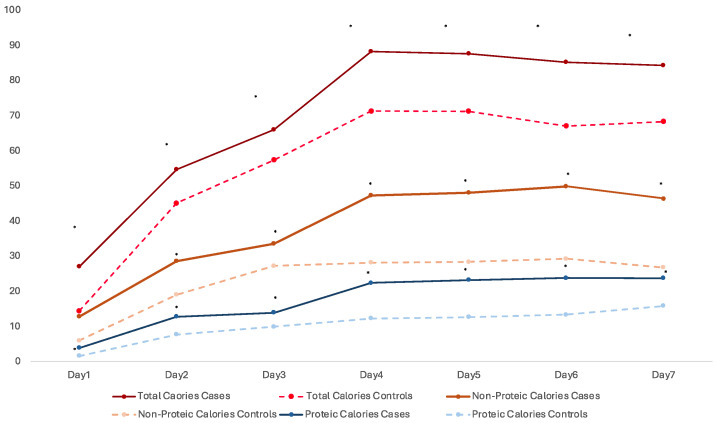
Mean daily actual parenteral caloric intake over the first 7 days of life in neonates who received total parenteral nutrition (PN), comparing cases and controls. Cases consistently received significantly higher total, non-protein, and protein caloric intake compared to controls. * *p* < 0.05.

We also performed a sub-analysis in neonates receiving total PN and newborns who received at least 90% of their total calories via PN. In these subgroups, the average daily intake of calories received through PN was compared between the two groups in the first week of life (Figure 1). This figure shows that the cases received a higher amount of calories and that the difference in the intake of calories (divided into total, protein, and non-protein) between the cases and controls in the first week of life was found to be statistically significant.

**Figure 2:**
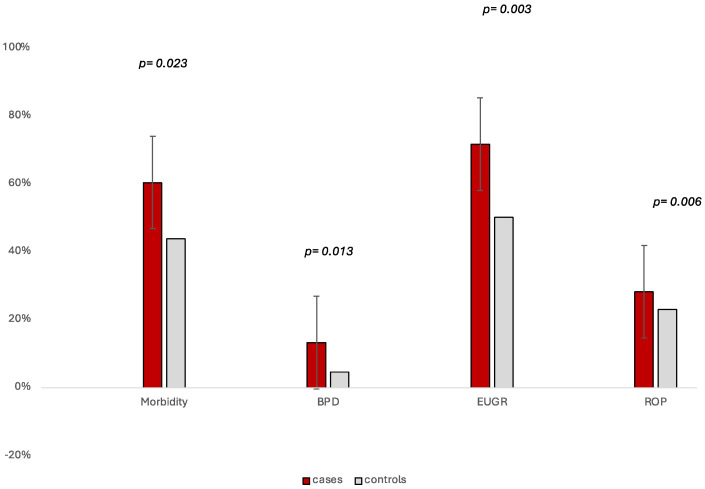
Incidence of selected neonatal morbidities between cases and controls, with 95% confidence intervals. Bars represent the percentage of neonates affected by overall morbidity (defined as at least one of the following morbidities: NEC, BPD, ROP, PVL, IVH, and sepsis), BPD, EUGR, and ROP. Error bars indicate the 95% confidence intervals calculated for each proportion in the respective group (cases: n = 53; controls: n = 359). Cases showed a significantly increased incidence of all outcomes (*p* < 0.05).

The case group showed an overall higher incidence of morbidity overall (60.4% vs. 43.7%; *p* = 0.023) (Figure 2). Figure 2 summarizes the main morbidities observed in the study population. In the case group, there was a significantly higher incidence of BPD (13.2% vs. 4.5%; *p* = 0.013), ROP (28.3% vs. 23.1%; *p* = 0.006), and EUGR (71.7% vs. 50.1%; *p* = 0.003), as well as a longer duration of non-invasive mechanical ventilation (10.83 vs. 6.33 days; *p* = 0.011), (Table 4). There were no significant differences between the two groups in terms of NEC stage of Bell ≥ 2, IVH ≥ 2, PLV, and sepsis (confirmed by a positive culture). The length of hospital stay was significantly higher in cases compared to controls (76.1 vs. 59.33 days; *p* = 0.001).

In Table 3, we reported the results of the multivariate analysis to evaluate the influence of covariates on the occurrence of RS. We found that fetal distress significantly influenced the occurrence of RS (Table 3). We also performed a binary logistic regression analysis including only those variables (all together) found to be statistically significant in the univariate analysis on the occurrence of RS (Supplementary Table S1).

We performed binary logistic regression analyses to evaluate the influence of covariates on the occurrence of the morbidities (ROP, EUGR, and BPD) that were statistically significant in the preliminary analysis.

Table 4 reports that RS, along with prolonged PN and FEF, significantly (*p* = 0.010) influenced the risk of EUGR (Table 4).

Table 5 shows that RS, along with GA and IMV in model I and along with BW and IMV in model II, significantly influenced the occurrence of ROP.

Neither of the two binary regression models evaluating the influence of covariates on the occurrence of BPD and NIV show that RS was not found to be statistically significant (Supplementary Tables S2 and S3).

## 4. Discussion

Fetal distress was an independent risk factor for the development of RS in VLBW neonates receiving early aggressive PN. In turn, the occurrence of RS in VLBW infants is significantly associated with the development of EUGR and ROP.

We adopted fetal distress as a proxy of placental insufficiency and as the association of both fetal Doppler abnormalities and acidosis at birth. These phenomena occurring immediately before the birth process could explain the reason why some preterm neonates are likely to develop RS after undergoing PN.

Acidosis at birth, combined with an abnormal fetal Doppler, has been defined as fetal distress, which represents not only a reduced oxygen supply but also a significant nutrient depletion from the mother to the fetus. In our opinion, this phenomenon may significantly contribute to the development of a baseline malnutrition condition that predisposes preterm neonates to RS occurrence. This happens during the abrupt interruption of nutrients in case of preterm delivery (during the fetal period, micronutrients are actively transferred through the placenta). This baseline depletion of nutrients includes the interruption of electrolyte transport (i.e., of phosphorus) from the mother to the fetus, resulting in an electrolyte imbalance at birth [9,10,13].

Among perinatal risk factors, fetal distress has been shown to be the predominant factor in the development of RS. Some studies have described that flowmetry abnormalities evaluated during pregnancy are the main indicator of placental dysfunction [8]. It has been suggested that flowmetry abnormalities could lead to a state of perinatal hypoxia, evidenced by acidosis on umbilical cord blood gas analysis [20,21]. Previous research has observed that the loss of fetal well-being acts as a perinatal risk factor for the development of RS [22,23]. Bonsante et al., in their prospective study including preterm neonates, evaluated the influence of a high intake of amino acids on calcium and phosphorus metabolism [8]. The authors introduced the definition of placental RS in order to indicate the important role of the placenta, particularly how its dysfunction results in a reduced nutrient supply, which clearly contributes to RS. However, their study focused on the nutritional aspects of RS and did not specify the concept of placental dysfunction or consider it in the multivariate statistical analysis. In line with our findings, prenatal stress was also considered a risk factor for RS in the observational study conducted by Boubred et al., which analyzed the incidence of RS in 48 infants with extremely low birth weights compared by their birth weight [24]. In this study, the explanation of the results was described in light of the pathophysiology outlined by Bonsante et al. [8]; they discussed that the increased incidence of RS in neonates was due to placental dysfunction. Nevertheless, the variable of placental dysfunction was not described and defined, they did not perform a multivariate statistical analysis to test its independent role, and they considered only neonates small for gestational age. Similarly, Pajak et al., in a retrospective study covering preterm newborns, found that neonates with a diagnosis of IUGR were likely to experience hypophosphatemia in the first week of life [25]. The explanation relied again on placental dysfunction that was responsible for a reduced supply of nutrients. However, the sample size was small, and placental dysfunction was not included in the multivariate analysis along with other clinical or nutritional confounding factors. In addition, in a case–control study investigating the main risk factors of developing RS, Igarashi et al. demonstrated that placental insufficiency, expressed by Doppler abnormality, was significantly associated with hypophosphatemia during the early postnatal period in VLBW infants [21]. Still, the variable of placental insufficiency by itself does not necessarily indicate fetal distress; moreover, it has not been compared to other nutritional variables in a regression analysis.

From our perspective, the development of RS in VLBW neonates could result from the combination of an initial micronutrient deficiency and the administration of aggressive parenteral nutrition. It has been widely demonstrated that high macronutrient intake induces a predominantly anabolic state, leading to the intracellular redistribution of electrolytes and lower levels of phosphate in serum blood in rapidly growing tissues [26,27]. In light of these biochemical mechanisms, previous evidence has established that RS in preterm neonates is linked to aggressive parenteral nutrition, specifically in terms of high intakes of amino acids in the presence of low electrolyte supply [8,28]. Despite this, those researchers focused on the amount of amino acids without separately examining the caloric intake (carbohydrates and lipids) other than the non-protein caloric intake.

Recently, in a large prospective cohort study conducted by Cormarck et al., it was observed that neonatal RS was common in neonates with an extremely low birth weight who received a high intake of macronutrients, including amino acids, carbohydrates, and lipids, but a reduced supply of micronutrients [14]. However, in the multivariate statistical analysis reported, only gestational age, weight, and male sex were used as confounding factors, without including any nutritional intakes. Moreover, those studies did not investigate the contribution of enteral nutrition. Taken together, those findings could depend on the local nutritional protocols adopted in NICUs. In a recently published study by Wright et al., the authors compared the occurrence of RS between neonates receiving early phosphate vs. those receiving standard phosphate [29]. They described that neonates receiving an early intake of phosphate were less likely to develop RS. However, they did not report the actual amount of phosphate received through PN between the two groups; moreover, they analyzed only preterm neonates with extremely low birth weights [29].

Overall, all the studies on the subject state that actual recommendations should be updated, particularly regarding the administration of micronutrients in the very first days of life. Despite this, we determined that neonates with RS did receive an increased intake of macronutrients and a higher amount of phosphorus. These findings reveal that a high intake of macronutrients, even when compensated by increased electrolyte intake via PN, still leads to the typical electrolyte abnormalities of the condition under examination (hypercalcemia with hypophosphatemia). Hence, we hypothesized that fetal distress may put neonates at risk of developing RS regardless of phosphorus intake. However, our study was not designed and powered to verify this hypothesis. We speculate that, in babies who are at risk, warranted phosphorus administration may support the process that had already begun once anabolism is induced.

Our results revealed a correlation between RS and EUGR. We hypothesized that IGF-1 plays a central role in the regulation of early postnatal growth. IGF-1 levels, after an initial drop at birth, increase steadily during the first eight weeks of life, ensuring proper extrauterine development [30]. Therefore, a reduction in IGF-1 levels during postnatal life, as occurs in RS, impairs the typical catch-up growth of the first weeks of life, leading to the onset of extrauterine growth retardation [30]. There are no studies in the literature linking EUGR with the development of RS. However, several studies have correlated EUGR with low levels of IGF-1 [30].

Our study found a significant association between RS and ROP, a retinal neovascular disorder affecting VLBW neonates and one of the most prevalent causes of blindness and childhood visual impairment worldwide. This condition is characterized by retinal microvascular degeneration associated with an arrest in the progressive vascularization of the peripheral retina, mainly due to exposure to an environment with more oxygen, resulting in retinal ischemia and the subsequent release of growth factors, leading to abnormal intravitreal neovascularization [31,32]. We speculate that the association between RS and ROP relies on the fact that the low concentration of phosphorus may negatively influence mitochondria and the energy metabolism of the ocular system. It has been widely established that adequate mitochondrial function is important in providing sufficient energy for organ development as it relates to decreasing oxidative stress via reactive oxygen species (ROS) [32,33]. Hypophosphatemia can alter oxygen transport in the retina vessels, which may result in markedly reduced levels of erythrocyte ATP and 2,3-DPG, which enhance the affinity of oxygen for hemoglobin, worsening ischemia. As previously described in diabetic patients regarding the pathogenesis of retinopathy, the interruption of optimal ATP production for any reason might lead to cell injury and thus vascular degeneration [31,32,33]. Moreover, premature birth has been associated with reduced levels of hormones, such as insulin growth factor 1 (IGF-1), which regulates retina growth [32,33,34]. IGF-1 levels in turn suppress the activation of endothelial cell proliferation by VEGF, leading to an arrest in the growth of blood vessels that is typical of phase I ROP pathogenesis [31,35]. Additionally, the already-induced ischemia upregulates the secretion of VEGF, which may be further elevated by the fact that, as the neonate grows, IGF-1 levels increase, thus exacerbating pathological revascularization [36]. Notably, there are no studies on preterm neonates in the literature correlating RS with an increased risk of ROP.

Despite being original, the results of our study should be interpreted considering some limitations. First of all, the retrospective design of our study carries inherent limitations. However, it is important to note that in order to mitigate this limitation, data collection and morbidity diagnoses were performed prospectively. Secondly, another potential limitation is that all the data were collected from patients admitted to a single center, the neonatal intensive care unit of Policlinico Umberto I, with a specific nutritional protocol. One potential limitation of the study is the relatively small sample size of the enrolled newborns, which may affect the generalizability of the findings. Results could differ in patients from other centers adopting different nutritional protocols. Finally, another potential limitation is that only some potential confounding factors, namely those that were significant in the univariate analysis, were used in multivariate statistical analyses, suggesting that different results could be observed if other clinical and nutritional factors were considered in the analysis. Statistical analysis was performed by a statistician who was unaware of this study’s aims.

Despite these limitations, our findings highlight the need for the early identification of neonates at risk of RS, even when current parenteral nutrition guidelines are followed. Future prospectives and multicenter studies are warranted to better define the role of perinatal risk factors in the development of RS.

## 5. Conclusions

Fetal distress is the predominant perinatal risk factor associated with the development of RS in preterm neonates. Moreover, we added evidence to the knowledge that RS is associated with clinical consequences, specifically EUGR and ROP, suggesting that neonatologists may need to ensure its early identification and closely follow up with at-risk babies. These observations highlight that the main risk factors of RS are modifiable factors that can be addressed in daily clinical practice in order to limit the development of complications. However, further studies are needed to confirm our findings.

## Figures and Tables

**Table 1:** Baseline characteristics of neonates included in this study.

	Cases	Controls	OR	*p*
	n = 53	n = 359		
Cesarean section, No. (%)	44 (83)	303 (86.8)	0.74 (0.34–1.62)	0.450
Twin pregnancy, No. (%)	14 (26.4)	111 (31.8)	0.77 (0.40–1.47)	0.431
Maternal age ≥ 35 years, No. (%)	33.8 (32.21 to 35.8)	34.07 (33.4 to 34.73)	-	0.762
Gestational diabetes, No. (%)	3 (5.7)	50 (13.9)	0.45 (1.13–1.51)	0.184
Pregnancy-induced hypertension, No. (%)	15 (28.3)	75 (20.9)	1.49 (0.78–2.60)	0.221
Thyroid disorders, No. (%)	5 (9.4)	47 (13.1)	0.69 (0.26–1.82)	0.593
Risk of infection ^1^, No. (%)	12 (22.6)	126 (35.1)	0.54 (0.27–1.06)	0.073
Placental Abruption, No. (%)	3 (5.7)	30 (8.4)	0.65 (0.19–2.30)	0.517
Antenatal corticosteroids ^2^, No. (%)	30 (56.6)	231 (64.7)	0.86 (0.32–1.78)	0.224
IUGR ^3^, No. (%)	11 (20.8)	53 (14.8)	1.51 (0.73–3.12)	0.260
Gestational age, weeks	28.79 (28.06 to 29.52)	29.68 (29.42 to 29.94)	-	0.016 *
ELBW ^4^, No. (%)	21 (39.6)	68 (19.2)	2.80 (1.38–4.72)	<0.001 *
Birth Weight, Z-score	−0.68 (−0.99 to −0.38)	−0.31 (−0.43 to −0.19)	-	0.029 *
Birth Length, Z-score	−0.58 (−0.98 to −0.18)	−0.25 (−0.46 to −0.11)	-	0.116
HC ^5^ at birth, Z-score	−0.36 (−0.66 to −0.07)	−0.08 (−0.05 to 0.22)	-	0.018
SGA ^6^, No. (%)	14 (26.4)	64 (17.8)	1.65 (0.84–3.22)	0.139
Male Gender, No. (%)	28 (52.8)	192 (53.8)	0.96 (0.54–1.71)	0.894
Apgar Score below 5, No. (%)	12 (22.6)	30 (8.8)	3.02 (1.43–6.36)	0.002 *
Fetal distress ^7^, No. (%)	6 (60.0)	4 (40.0)	8.70 (2.3–31.6)	0.002 *
Surfactant, No. (%)	26 (49.1)	143 (39.8)	1.45 (0.81–2.59)	0.202
Caffeine, No. (%)	49 (92.5)	295 (82.2)	2.65 (0.92–7.62)	0.061

Notes. All continuous variables are expressed as means (95% CI) unless otherwise specified. ^1^ Risk of infection: at least one out of maternal fever (temperature > 37.5 °C), chorioamnionitis, maternal urinary tract infection (UTI), prolonged rupture of membranes without adequate maternal antibiotic prophylaxis (PAI), positive vaginal–rectal swab for Group B Streptococcus without adequate PAI, and a previous child with invasive GBS disease; ^2^ antenatal corticosteroids: prepartum administration of two doses of 12 mg of steroids in less than 24 h; ^3^ IUGR: intrauterine growth restriction; ^4^ ELBW: extremely low birth weight; ^5^ HC: head circumference; ^6^ SGA: small for gestational age; ^7^ fetal distress: fetal Doppler abnormalities and acidosis at birth (umbilical cord pH < 7.2). * *p* < 0.05.

**Table 2:** Nutritional baseline characteristics of neonates included in this study.

	Cases	Controls	OR	*p*
	n = 53	n = 359		
MEF ^1^ 0–7 days, No. (%)	23 (43.4)	228 (63.5)	0.44 (0.24–0.79)	0.005 *
FEF ^2^ 7 days, No. (%)	7 (13.2)	98 (27.3)	0.40 (0.17–0.92)	0.028 *
EN ^3^ 0–7 days, g/kg/day	127.0 (82.8 to 171.3)	205.4 (184.0 to 226.8)	-	0.008 *
EN 0–3 days, g/kg/day	30.7 (21.2 to 43.1)	40.1 (28.1 to 50.3)	-	0.181
EN 4–7 days, g/kg/day	97.3 (84.8 to 134.4)	143.4 (131.6 to 159.1)	-	0.041 *
Prolonged PN ^4^, No. (%)	42 (79.2)	208 (57.9)	2.77 (1.38–5.56)	0.003 *
PN 0–7 days, g/kg/day	673.9 (609.2 to 738.7)	481.2 (452.0 to 51.5)	-	<0.001 *
PN 0–3 days, g/kg/day	267.4 (94.4 to 300.5)	200.5 (117.7 to 267.9)	-	<0.001 *
PN 4–7 days, g/kg/day	406.5 (388.7 to 456.2)	280.6 (192.2 to 334.7)	-	<0.001 *
Total Calories PN ^5^, g·kg·wk^−1^	312.2 (257.1–367.3)	186.3 (166.4–206.2)	-	<0.001 *
Non-proteic Cal PN ^6^, g·kg·wk^−1^	231.9 (186.6–277.2)	133.8 (118.2–149.5)	-	<0.001 *
Proteic Cal PN ^7^, g·kg·wk^−1^	94.1 (78.8–109.4)	55.6 (50.5–60.7)	-	<0.001 *
Calcium PN ^8^, g·kg·wk^−1^	312.2 (257.1–367.3)	186.3 (166.4–206.2)	-	<0001 *
Phosphorus PN ^9^, g·kg·wk^−1^	334.0 (273.3–394.7)	239.0 (162.1–315.9)	-	0.355
Calcium/Phosphorus PN ^10^	28.0 (52.8)	147.0 (40.9)	1.61 (1.43–1.98)	1.020

Notes. Continuous variables are expressed as means (95% CI) unless otherwise specified. ^1^ MEF: minimal enteral feeding in the first week > 70 mL/kg; ^2^ FEF: full enteral feeding (120 kcal/kg/day); ^3^ EN: enteral nutrition; ^4^ prolonged PN: parenteral nutrition > 7 days; ^5^ total calories: calories received within the first week of life; ^6^ non-protein calories: non-protein calories received within the first week of life; ^7^ protein calories PN: protein calories received within the first week of life from PN; ^8^ calcium: calcium received within the first week of life; ^9^ phosphorus PN: phosphorus received within the first week of life; ^10^ calcium/phosphorus PN: calcium/phosphorus ratio > 1, received during the first week of life. * *p* < 0.05.

**Table 3:** Binary logistic regression analysis to evaluate the influence of baseline risk factors on RS.

Variables	β	Wald	*p*-Value	Odds Ratio (OR)	95 C.I for OR	
					Lower	Upper
***Model I***						
GA ^1^	0.037	0.008	0.927	1.038	0.469	2.299
Male sex	0.013	0.002	0.968	1.013	0.550	1.864
Fetal distress ^2^	0.798	4.818	0.028 *	2.220	1.089	4.527
PN Calories ^3^	0.512	1.181	0.277	1.668	0.663	4.200
PN Calcium ^4^	0.420	0.642	0.423	1.521	0.545	4.245
PN Phosphorus ^5^	0.005	0.000	0.993	1.005	0.337	2.996
MEF ^6^	−0.150	0.169	0.681	0.861	0.422	1.758
***Model II***						
ELBW ^7^	0.087	0.043	0.835	1.091	0.481	2.476
Male sex	0.016	0.003	0.959	1.016	0.551	1.873
Fetal distress	0.795	4.952	0.026 *	2.214	1.099	4.458
PN Calories	0.404	0.580	0.446	1.498	0.529	4.240
PN Calcium	0.001	0.000	0.999	1.001	0.338	2.963
PN Phosphorus	0.134	0.130	0.719	0.874	0.421	1.817
MEF	0.087	0.043	0.835	1.091	0.481	2.476
***Model III***						
GA	0.276	0.435	0.509	1.318	0.581	0.276
Male sex	0.100	0.095	0.758	1.105	0.584	0.100
Fetal distress	0.831	4.824	0.028 *	2.295	1.093	0.831
Non-proteic calories ^8^	0.955	3.346	0.067	2.598	0.934	7.224
Proteic calories ^9^	0.522	0.898	0.343	1.686	0.572	4.963
PN Calcium	0.001	0.000	0.999	1.001	0.309	0.001
PN Phosphorus	−0.184	0.204	0.651	0.832	0.376	−0.184
MEF	0.299	0.220	0.639	1.349	0.387	0.299
***Model IV***						
ELBW	−0.250	0.234	0.628	0.779	0.283	2.142
Male sex	0.091	0.079	0.779	1.096	0.579	2.073
Fetal distress	0.785	4.489	0.034 *	2.192	1.061	4.531
Non-proteic calories	0.944	3.199	0.074	2.569	0.914	7.225
Proteic calories	0.531	0.910	0.340	1.700	0.571	5.057
PN Calcium	0.346	0.293	0.588	1.414	0.404	4.953
PN Phosphorus	0.114	0.037	0.847	1.021	0.352	3.563
MEF	−0.256	0.385	0.535	0.774	0.344	1.739

Notes. ^1^ GA: gestational age ≤ 30; ^2^ fetal distress: abnormal blood flow and/or umbilical pH < 7.2; ^3^ calories PN: amount of calories received by PN in the first week of life > 450 kcal/kg/day. ^4^ Calcium PN: amount of calcium received by PN in the first week of life > 300 mg/kg/day. ^5^ Phosphorus PN: amount of phosphorus received by PN in the first week of life > 250 mg/kg/day; ^6^ MEF: minimal enteral feeding > 70 mL received in the first week of life; ^7^ ELBW: birth weight ≤ 1000 g; ^8^ non-protein calories: received by PN in the first week of life > 410 Kcal/kg; ^9^ protein calories received by PN in the first week of life > 75 Kcal/kg. * *p* < 0.05.

**Table 4:** Binary logistic regression analysis to evaluate covariates influencing the occurrence of EUGR.

Variables	β	Wald	*p*-Value	Odds Ratio (OR)	95 C.I for OR	
					Lower	Upper
*Model I*						
GA ^1^	−1.039	15.839	0.000 *	0.354	0.212	3.135
Male sex	0.060	0.073	0.786	1.062	0.688	1.714
Fetal distress ^2^	−0.095	0.099	0.753	0.909	0.502	1.287
IUGR ^3^	1.264	12.735	0.000 *	3.539	1.768	7.967
Prolonged PN ^4^	1.138	9.677	0.002 *	3.120	1.523	4.403
FEF ^5^	0.135	0.118	0.731	1.145	0.529	3.338
RS ^6^	0.913	6.585	0.010 *	2.493	1.241	4.155
*Model II*						
ELBW ^7^	0.589	4.335	0.037	1.801	1.035	3.135
Male sex	0.112	0.267	0.605	1.119	0.730	1.714
Fetal distress	−0.337	1.256	0.262	0.714	0.396	1.287
IUGR	1.390	15.832	0.000 *	4.017	2.025	7.967
Prolonged PN	0.794	5.111	0.024 *	2.212	1.111	4.403
FEF	0.463	1.489	0.222	1.588	0.756	3.338
RS	0.734	4.352	0.037 *	2.084	1.045	4.155

Notes. ^1^ GA: gestational age ≤ 30 weeks; ^2^ fetal distress: abnormal blood flow and/or umbilical pH < 7.2; ^3^ IUGR: intrauterine growth restriction; ^4^ prolonged PN: PN > 7 days; ^5^ FEF: achievement of full enteral feeding at 7 days of life; ^6^ RS: refeeding Syndrome; ^7^ ELBW: extremely low birth weight < 1000 g. * *p* < 0.05.

**Table 5:** Binary logistic regression analysis to evaluate covariates influencing the occurrence of ROP.

Variables	β	Wald	*p*-Value	Odds Ratio (OR)	95 C.I for OR	
					Lower	Upper
*Model I*						
GA ^1^	2.256	20.440	0.000 *	9.545	3.589	3.589
Male sex	−0.382	1.666	0.197	0.683	0.382	0.382
Fetal distress ^2^	−0.646	2.581	0.108	0.524	0.238	0.238
IUGR ^3^	0.349	0.670	0.413	1.418	0.614	0.614
IMV ^4^	1.001	11.198	0.001 *	2.720	1.514	1.514
RS ^5^	1.171	7.287	0.007 *	2.764	1.321	1.321
*Model II*						
ELBW ^6^	1.513	22.922	0.000 *	4.541	2.444	8.437
Male sex	−0.366	1.531	0.216	0.693	0.388	1.238
Fetal distress	−0.565	1.890	0.169	0.569	0.254	1.272
IUGR	−0.310	0.560	0.454	0.733	0.325	1.652
IMV	0.953	9.310	0.002 *	2.593	1.406	4.782
RS	0.952	6.482	0.011 *	2.590	1.245	5.390

**Notes.** ^1^ GA: gestational age ≤ 30 weeks; ^2^ fetal distress: abnormal blood flow and/or umbilical pH < 7.2.; ^3^ IUGR: intrauterine growth restriction; ^4^ IMV: invasive mechanical ventilation; ^5^ refeeding syndrome; ^6^ ELBW: extremely low birth weight < 1000 g. * *p* < 0.05.

## Data Availability

The datasets analyzed during the current study are available from the corresponding author upon reasonable request, due to privacy.

## Supplementary Materials

The following supporting information can be downloaded at: https://www.mdpi.com/article/10.3390/nu17091417/s1, Table S1: Binary logistic regression analysis to evaluate covariates influencing the occurrence of BPD; Table S2: Binary logistic regression analysis to evaluate covariates influencing the duration of non-invasive mechanical ventilation. Table S3: Binary logistic regression analysis to evaluate covariates influencing the duration of non-invasive mechanical ventilation.

## Abbreviations

BPD	Bronchopulmonary Dysplasia
BW	Birth Weight
CRIBB	Clinical Risk Index for Babies
CVO	Central Vascular Access
EUGR	Extrauterine Growth Restriction
EN	Enteral Nutrition
FEF	Full Enteral Feeding
GA	Gestational Age
HC	Head Circumference
IGF-1	Insulin-Like Growth Factor 1
IMV	Invasive Mechanical Ventilation
IVH	Intraventricular Hemorrhage
MEF	Minimal Enteral Feeding
MRI	Magnetic Resonance Imaging
NEC	Necrotizing Enterocolitis
NICU	Neonatal Intensive Care Unit
NIV	Non-Invasive Ventilation
PN	Parenteral Nutrition
PICC	Peripherally Inserted Central Catheter
PLV	Periventricular Leukomalacia
ROP	Retinopathy of Prematurity
RS	Refeeding Syndrome
VLBW	Very Low Birth Weight
