# Supplementary material for: Fetal Distress as a Determinant for Refeeding Syndrome in Preterm Neonates
Source: Nutrients. 2025 Apr 23;17(9):1417. doi: 10.3390/nu17091417 (PMC12073788; doi:10.3390/nu17091417)
Supplement: Supplementary file 1 [file nutrients-17-01417-s001.zip › nutrients-3536936-supplementary.pdf]

Supplementary Materials

Supplementary Table S1. Binary logistic regression analysis to evaluate the influence of variables found to be statistically significant in the univariate analysis on the occurrence of RS.

Variables	β	Wald	*p value*	Odds ratio (OR)	95 C.I for OR	
					Lower	Upper
GA[1]	0.017	0.002	0.965	1.017	0.474	2.185
ELBW[2]	-0.094	0.047	0.828	0.911	0.392	2.113
Apgar Score[3]	0.660	2.574	0.109	1.935	0.864	4.333
Fetal distress [4]	1.385	3.763	0.05*	3.994	0.986	16.183
PN Calories [5]	0.147	0.069	0.792	1.159	0.387	3.469
Non-proteic calories[6]	0.857	2.610	0.106	2.355	0.833	6.658
Proteic calories[7]	0.476	0.647	0.421	1.610	0.505	5.137
PN Calcium[8]	0.409	0.669	0.413	1.506	0.565	4.016
MEF[9]	-0.100	0.070	0.791	0.905	0.431	1.898
FEF[10]	-0.245	0.243	0.622	0.783	0.296	2.071

Notes. [1]GA: gestational age ≤ 30; [4]ELBW: extremely low birth weight; [3]Apgar Score below 5; [4]Fetal distress: fetal doppler abnormalities and acidosis at birth (umbilical cord pH < 7.2); [5]Calories PN: amount of calories received by PN in the first week of life >450 kcal/kg/day; [6]Non-proteic calories: received by PN in the first week of life > 410Kcal/kg; [7]Proteic calories received by PN in the first week of life >75 Kcal/kg; [8]Calcium PN: amount of calcium received by PN in the first week of life >300 mg/kg/day; [9]MEF: Minimal enteral feeding >70 mL received in the first week of life; [10]FEF: full enteral feeding (120 kcal/kg/day).

Supplementary Table S2. Binary logistic regression analysis to evaluate covariates influencing the occurrence of BPD.

1.1 Model I

Variables	β	Wald	*p value*	Odds ratio (OR)	95 C.I for OR	
					Lower	Upper
GA[1]	170.90	0.000	0.995	41.352	12.000	56.99
Male sex	0.453	0.855	0.355	1.573	0.602	4.106
Fetal distress [2]	0.706	1.846	0.174	2.026	0.732	5.607
IUGR [3]	0.325	0.236	0.627	10.384	0.373	5.135
IMV [4]	2.326	13.147	0.000*	10.238	2.912	35.997
RS [5]	0.938	3.025	0.082	2.554	0.888	7.350

Notes. [1] GA: gestational age ≤ 30 weeks; [2] Fetal distress:abnormal blood flow and/or umbilical pH <7.2. [2] Fetal distress: abnormal blood flow and/or umbilical pH <7.2; [3] IUGR: intrauterine growth restriction; [4] IMV: invasive mechanical ventilation; [5] Refeeding Syndrome.

1.2 Model II

Variables	β	Wald	*p value*	Odds ratio (OR)	95 C.I for OR	
					Lower	Upper
ELBW[2]	1.884	9.660	0.002	6.582	2.006	21.596
Male sex	0.425	0.721	0.396	1.530	0.573	4.080
Fetal distress [2]	0.739	1.929	0.165	2.094	0.738	5.943
IUGR [3]	-0.209	0.099	0.753	0.811	0.220	2.988
IMV [4]	2.157	10.487	0.001	8.648	2.343	31.910
RS [5]	1.010	3.399	0.065	2.745	0.938	8.032

Notes. [1] ELBW: extremely low birth weight <1000 g; [2] Fetal distress:abnormal blood flow and/or umbilical pH <7.2. [2] Fetal distress: abnormal blood flow and/or umbilical pH <7.2; [3] IUGR: intrauterine growth restriction; [4] IMV: invasive mechanical ventilation; [5] Refeeding Syndrome.

Supplementary Table S3. Binary logistic regression analysis to evaluate covariates influencing the duration of non-invasive mechanical ventilation.

2.1 Model I

Variables	β	Wald	*p value*	Odds ratio (OR)	95 C.I for OR	
					Lower	Upper
GA[1]	1.160	16.054	0.000	3.192	1.809	5.630
Male sex	0.601	4.781	0.029	1.824	1.064	3.128
Fetal distress [2]	0.822	2.680	0.102	2.274	.850	6.081
IUGR [3]	-0.672	4.133	0.042	0.510	0.267	0.976
RS [4]	0.006	0.000	0.990	1.006	0.425	2.377

Notes. [1] GA: gestational age ≤ 30 weeks; [2] Fetal distress:abnormal blood flow and/or umbilical pH <7.2. [3] IUGR: intrauterine growth restriction; [4] RS: Refeeding Syndrome.

2.2 Model II

Variables	β	Wald	*p value*	Odds ratio (OR)	95 C.I for OR	
					Lower	Upper
ELBW[2]	4.277	0.039	2.143	1.041	4.411	4.277
Male sex	4.151	0.042	1.731	1.021	2.933	4.151
Fetal distress [2]	4.211	0.040	2.771	1.047	7.336	4.211
IUGR [3]	9.324	0.002	0.371	0.196	0.701	9.324
RS [4]	0.007	0.935	1.036	0.445	2.411	0.007

Notes. [1] ELBW: extremely low birth weight < 1000g; [2] Fetal distress:abnormal blood flow and/or umbilical pH <7.2. [3] IUGR: intrauterine growth restriction; [4] RS: Refeeding Syndrome.
